# Supplementary material for: In Vitro, In Vivo, and In Silico Analyses of Molecular Anti-Pigmentation Mechanisms of Selected Thai Rejuvenating Remedy and Bioactive Metabolites
Source: Molecules. 2023 Jan 18;28(3):958. doi: 10.3390/molecules28030958 (PMC9920523; doi:10.3390/molecules28030958)
Supplement: Supplementary file 1 [file molecules-28-00958-s001.zip › molecules-2092317-supplementary.pdf]

In vitro, In vivo, and In Silico Analyses of Molecular Anti-pigmentation mechanisms of Selected Thai Rejuvenating Remedy and Bioactive Metabolites.

Sukanya Dej-adisai [1,] *, Nitinant Koyphokaisawan [1], Chatchai Wattanapiromsakul [1], Wanlapa Nuankaew [2], Tong Ho Kang [2] and Thanet Pitakbut [3]

[1]Department of Pharmacognosy and Pharmaceutical Botany, Faculty of Pharmaceutical Sciences, Prince of Songkla University, Hat Yai, Songkhla 90112, Thailand; nitinant.kp@gmail.com (NK); chatchai.w@psu.ac.th (CW.)

[2]Department of Oriental Medicinal Biotechnology, Graduate School of Biotechnology, College of Life Sciences, Kyung Hee University, Gyeonggi-do 17104, Republic of Korea; wanlapa.nuankaew@gmail.com (WN); panjae@khu.ac.kr (TK.)

[3]Pharmaceutical Biology, Department of Biology, Faculty of Sciences, Friedrich-Alexander-Universität Erlangen-Nürnberg (FAU), Staudtstr. 5, 91058 Erlangen, Germany; E-mails: thanet.pitakbut@fau.de (TP.)

*Correspondence: sukanya.d@psu.ac.th; Tel.: +66-74-288-888; Fax: +66-74-288-891

Supplementary file

Figure S1. Promoting melanogenesis effect of low and high concentrations of α-MSH at 0.25 nM and 10 nM.

Figure S2. A linear relationship between murine melanoma (B16F1) cells intracellular melanin content
and zebrafish larvae assays.

Figure S3. Phylogenetic analysis of eighteen tyrosinases amino acids sequences.

Figure S4. Docking validation protocol for tyrosinase.

Figure S5. 2D molecular interaction between glabridin and tyrosinases from mushroom, murine, and zebrafish.

Figure S6. Overlaying docking results of glabridin and its derivatives on mushroom tyrosinase.

Figure S7. Overlaying docking results of glabridin and its derivatives on murine tyrosinase.

Figure S8. Overlaying docking results of glabridin and its derivatives on zebrafish tyrosinase.

Figure S9. Protein structural alignment between human and zebrafish MC1R.

FigureS10. Docking validation protocol for zebrafish MC1R.

FigureS11. Combining docking results between zebrafish MC1R and glabridin derivatives.

FigureS12. Combining docking results between zebrafish MC1R and ethyl-p-methoxycinnamate derivatives.

Table S1. Anti-mushroom tyrosinase screening of sixty-two Thai rejuvenating remedies.

Table S2. Intercellular melanin content of B16F1 cells treated with α-MSH, positive controls (arbutin, kojic acid, and *A. lakoocha* wood, water extract), and test samples (crude, hexane, ethyl acetate, and ethanol extracts of the selected remedy).

Table S3. Cell viability test of arbutin up to 200 μg/ml concentration.

Table S4. Anti-zebrafish pigmentation of all samples.

Table S5. Accession numbers of all tyrosinase amino acid sequences used in this study.

Table S6. Chemical list in the PubChem database.

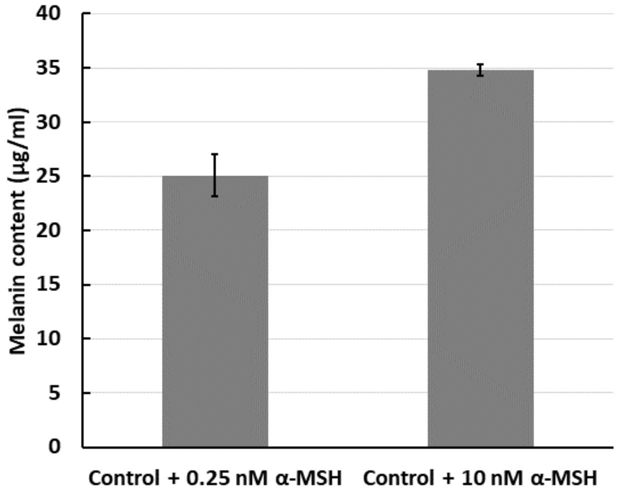

Figure S1 Promoting melanogenesis effect of low and high concentrations of α-MSH at 0.25 nM and 10 nM

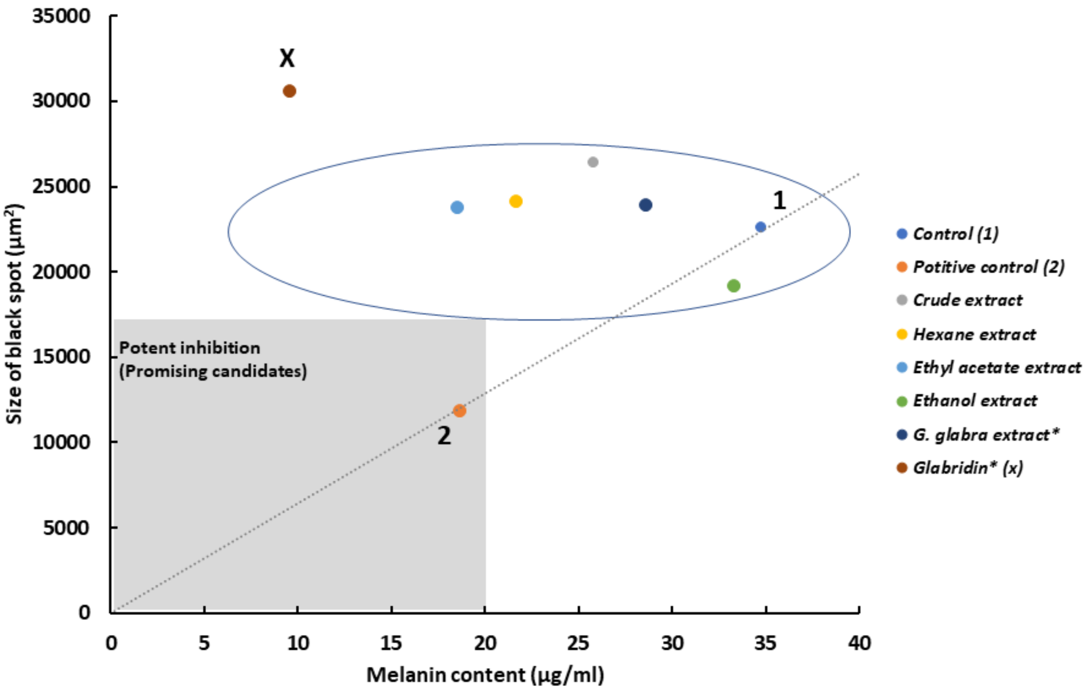

Figure S2 A linear relationship between murine melanoma (B16F1) cells intracellular melanin content and zebrafish larve assays obtained from positive controls (Arbutin, for the melanin content assay, and PTU, for the zebrafish larve assay), remedy extracts (crude,, hexane, ethyl acetate, ethanol, and water extracts and active ingredients (*G. glbra* extract, and glabridin, X). X indicates a sample outside the cluster.

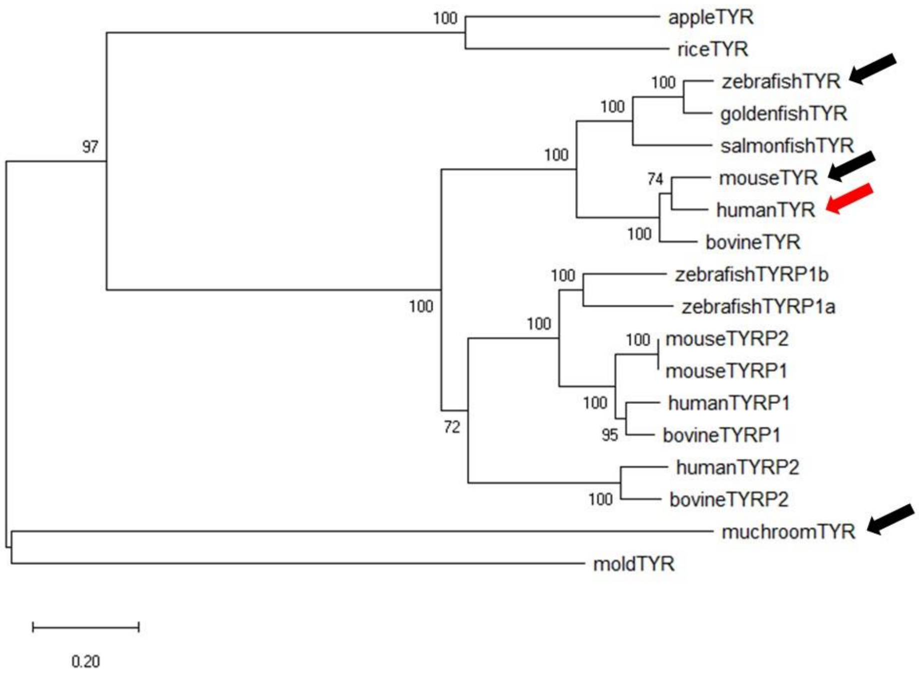

Figure S3 Phylogenetic analysis of eighteen tyrosinase (TYR) and tyrosinase-like protein (TYRP). Red arrow indicates human tyrosinase, while black arrows indicate tyrosinase from orgainsms used in this study. Multiple alignments are constructed using cluster W tool and phylogenetic analysis is done by Minimum Evolution method from MEGA-X software (Version 10.0.4). Bootstrap values are presented on the branch based on 1,000 pseudoreplicates.

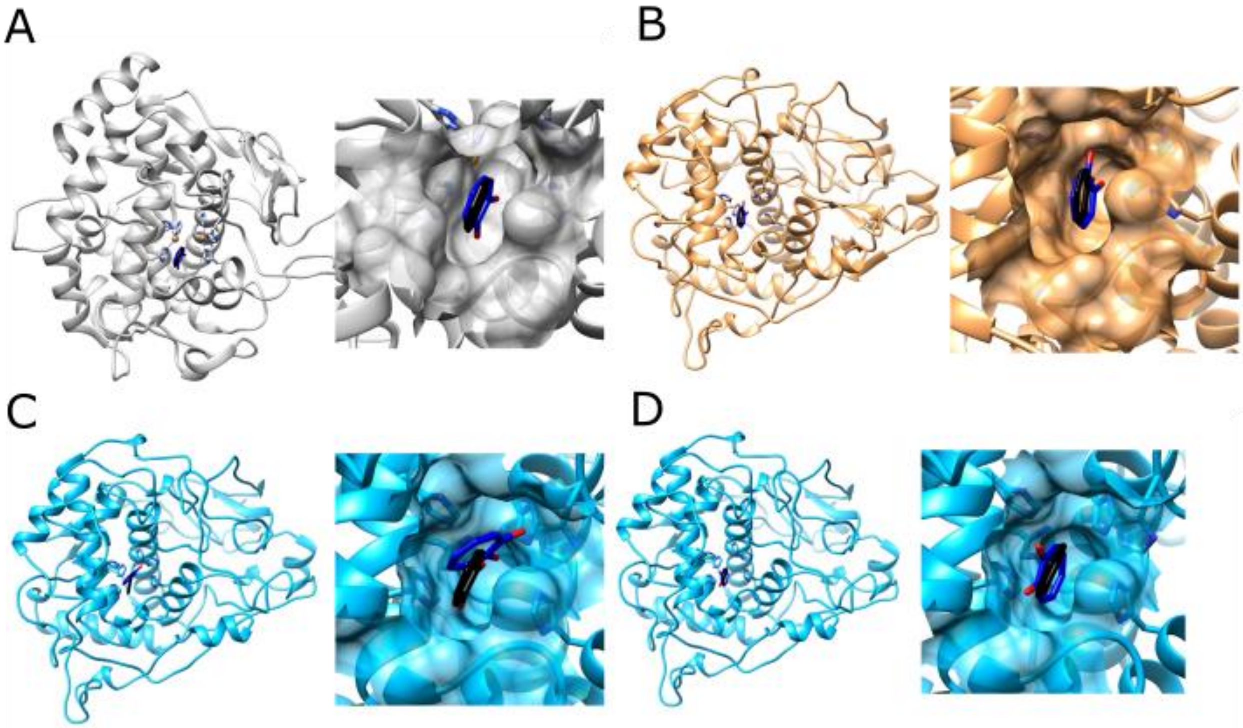

Figure S4. Docking protocol validation. (A) Validation docking protocol for mushroom tyrosinase (gray). Crystal tropolone (blue, native ligand) is re-docked back to its original pose (black). The RMSD comparing re-docked and original poses is used to validate the docking protocol, and the RMSD between re-docked and original tropolone is 0.981 Å. (B) Validation docking protocol for murine tyrosinase (brown). Like earlier, crystals tropolone (blue) is re-docked back to the original pose (black). The RMSD comparing re-docked and original poses is used to validate the docking protocol, and the RMSD between re-docked and original tropolone is 2.465Å. (C) Finally, validation protocol for zebrafish tyrosinase (light blue). Again, crystal tropolone (blue, native ligand) is re-docked back to its original pose (black). The RMSD comparing re-docked and original poses is used to validate the docking protocol, and the RMSD between re-docked and original tropolone is 2.326 Å. However, the structural alignment is not satisfied. Therefore, another re-docking pose has been selected, as shown in (D). However, the RMSD is higher than 2.500 Å. Even if this is the case, the structural alignment between the original and re-docked structure is more satisfying than earlier.

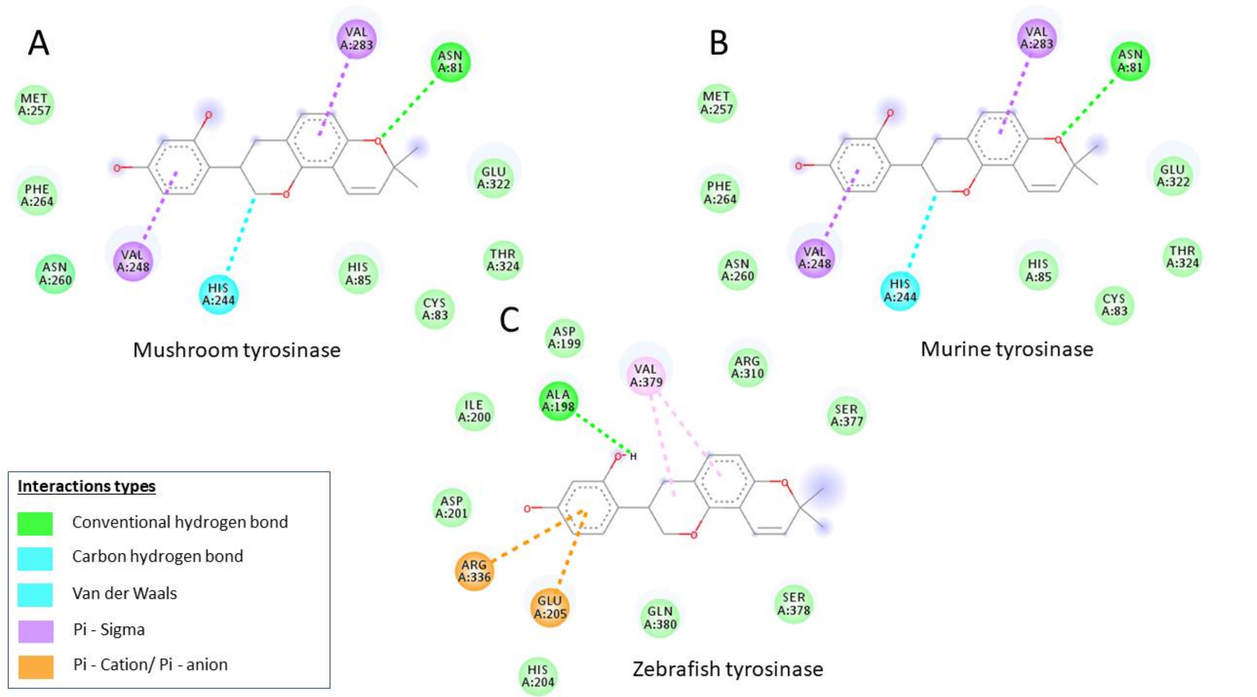

Figure S5. 2D molecular interaction between glabridin and tyrosinases from mushroom, murine, and zebrafish. (A) interaction diagram between glabridin and mushroom tyrosinase. (B) Interaction diagram between glabridin and murine tyrosinase. (C) Interaction diagram between glabridin and zebrafish tyrosinase.

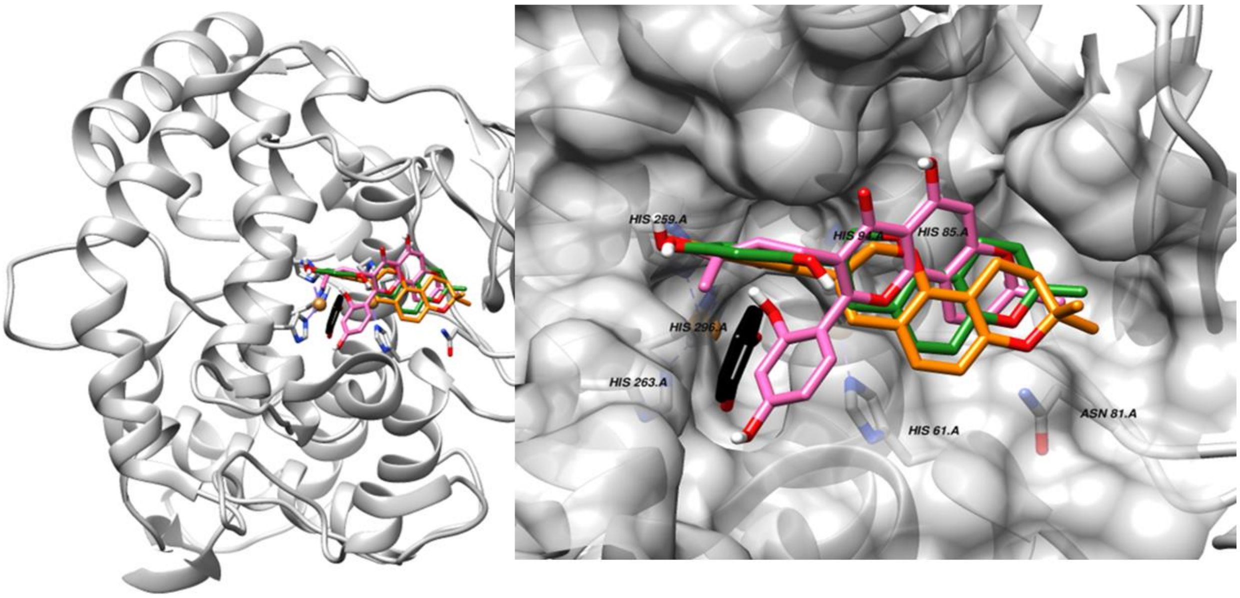

Figure S6. Overlaying docking results of glabridin (green) and its derivatives, 3'',4''-dihydro-glabridin (orange), and morusone (pink), on mushroom tyrosinase (grey).

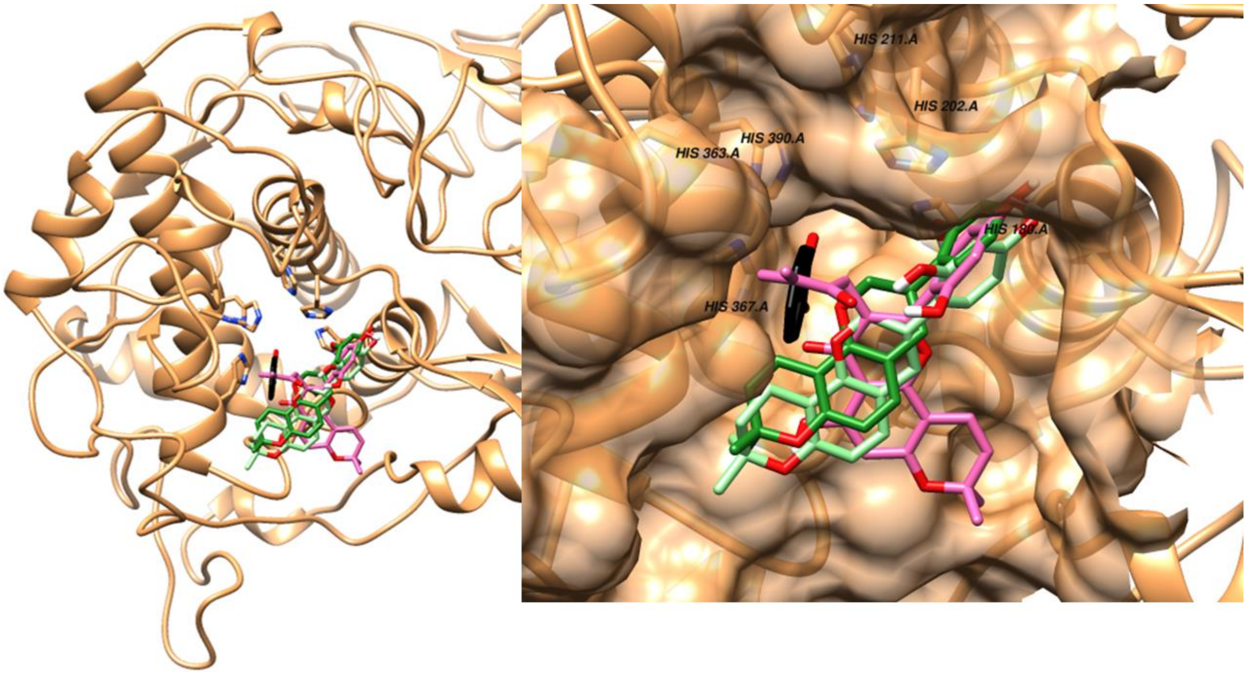

Figure S7. Overlaying docking results of glabridin (green) and its derivatives, 3′′,4′′-dihydro-glabridin (orange), and morusone (pink), on murine tyrosinase (grey).

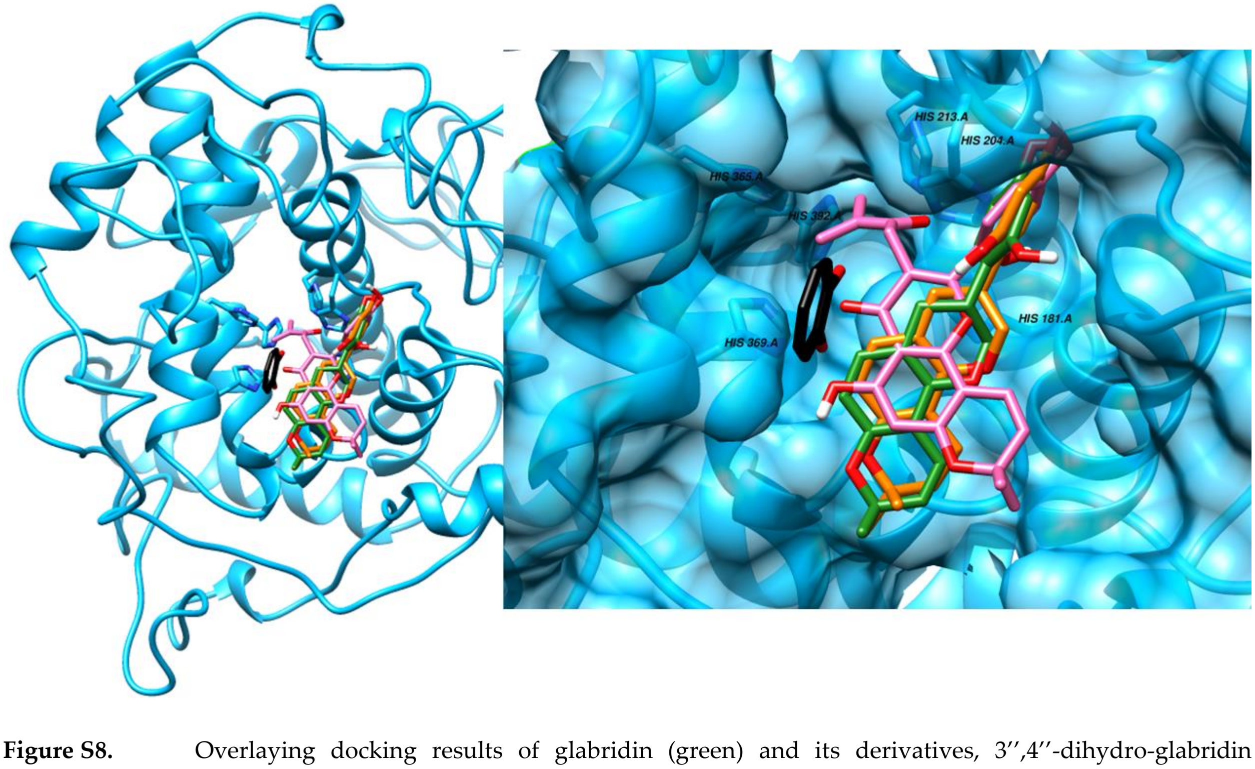

Figure S8. Overlaying docking results of glabridin (green) and its derivatives, 3′′,4′′-dihydro-glabridin (orange), and morusone (pink), on zebrafish tyrosinase (grey).

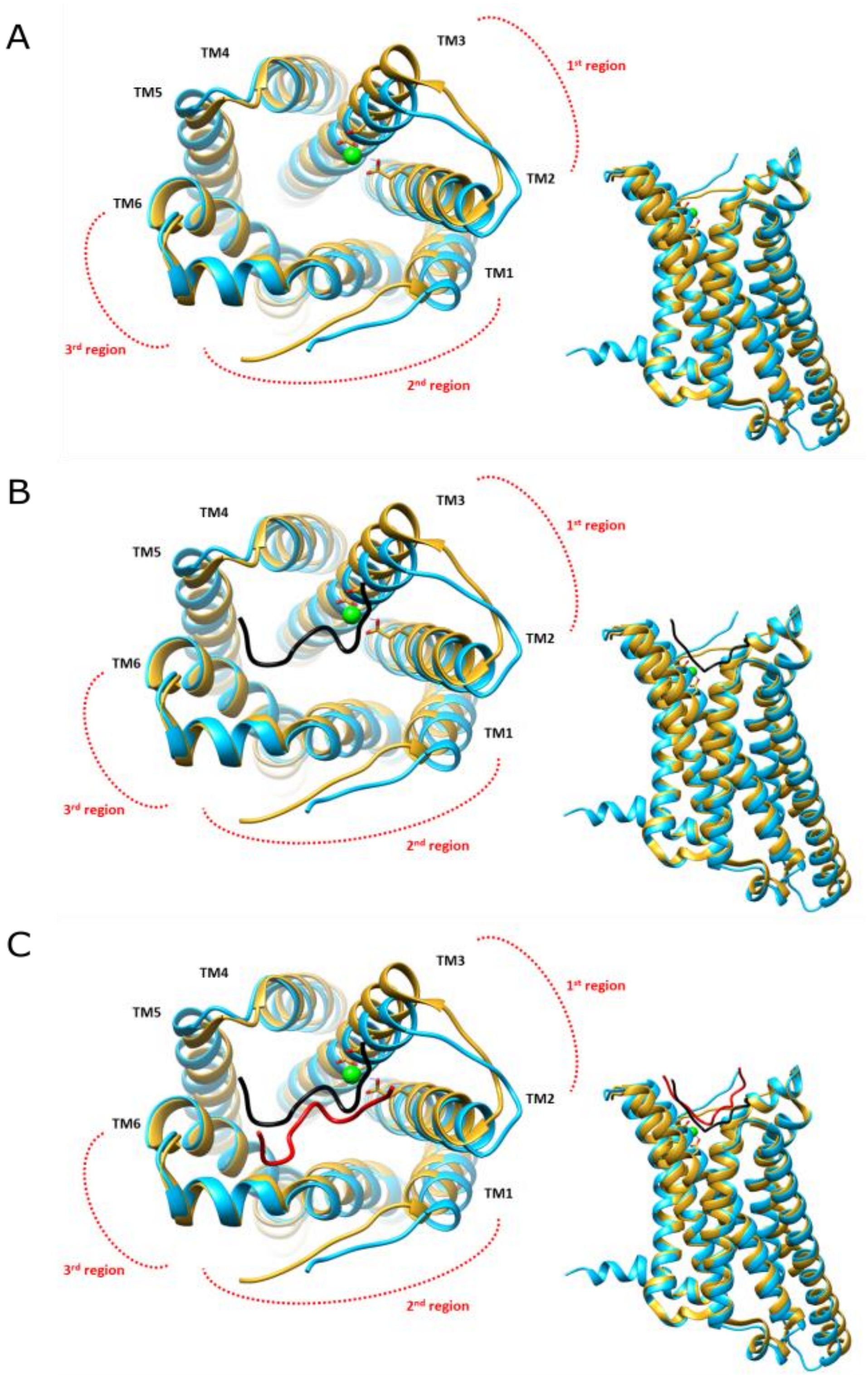

Figure S9. Protein structural alignment between human and zebrafish MC1R. (A) Superposition of protein structures between human MC1R presented in gold and zebrafish in blue. (B) Superposition of human MC1R presented in gold and a native MSH in red color and zebrafish in blue. (C) Superposition of native MSH, black, laid at a binding pocket on human MC1R receptor, gold, and a docked MSH, red, in a predicted binding site on zebrafish MC1R receptor, blue. TM1 to TM6 represent six transmembrane domains of all three essential regions on the MC1R receptor.

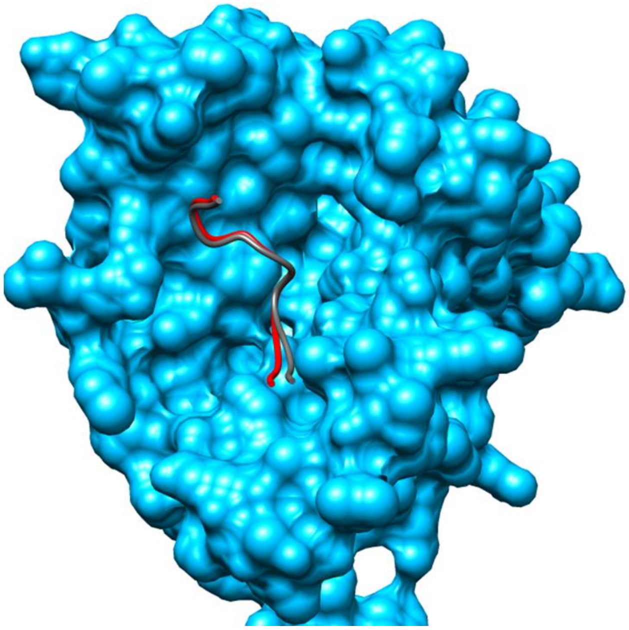

Figure S10. Docking protocol validation for zebrafish MC1R (blue) by re-docked α-MSH (grey) back to its purposed pose (red). The RMSD comparing re-docked and original poses is used to validate the docking protocol, and the RMSD between re-docked and original α-MSH is 1.118 Å.

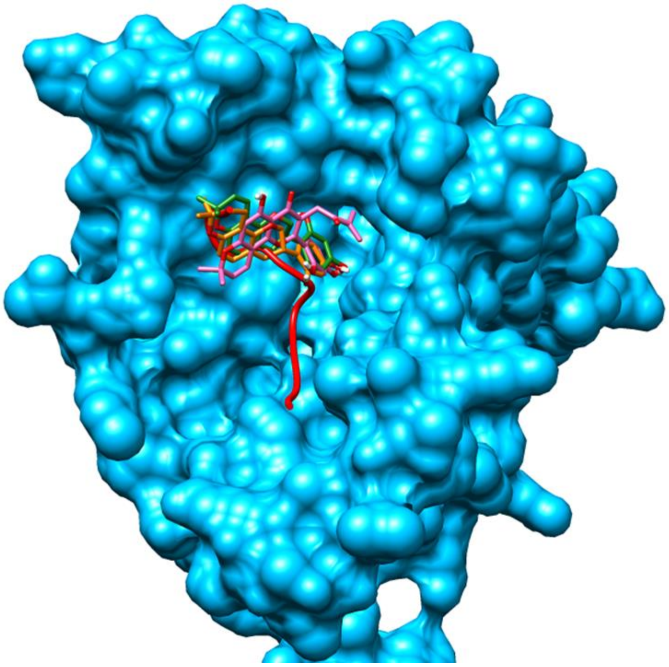

FigureS10. Combining docking results between zebrafish MC1R (blue) and glabridin derivatives, including glabridin (green), 3′′,4′′-dihydro-glabridin (orange), and morusone (pink), while α-MSH as a natural ligand represents in red.

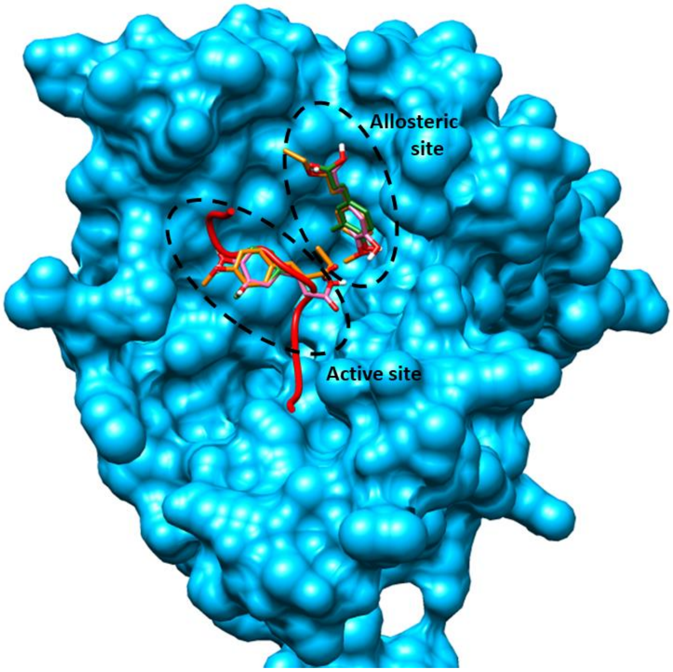

FigureS11. Combining docking results between zebrafish MC1R (blue) and ethyl-*p*-methoxycinnamate, including ethyl-*p*-methoxycinnamate (green), 3-methyl-4-hydroxycinnamic acid (orange), and caffeic acid (pink), while α-MSH as a natural ligand represents in red.

Table S1. Anti-mushroom tyrosinase Screening of sixty-two Thai rejuvenating remedies with 80% ethanolic extracts.

No.	Thai rejuvenating remedies (Sample code)	Tyrosinase inhibitory activity	
		Tyrosinase inhibition at 20 μg/ml (%)	IC_{50} values (μg/ml)
1	No. 1	18.39 ± 1.49	-
2	No. 2	36.75 ± 1.06	-
3	No. 3	15.94 ± 2.46	-
4	No. 4	17.00 ± 0.47	-
5	No. 5	24.42 ± 1.19	-
6	No. 6	57.30 ± 1.64	-
7	No. 7	31.77 ± 0.43	-
8	No. 8	16.11 ± 1.68	-
9	No. 9	7.05 ± 2.03	-
10	No. 10	23.62 ± 3.14	-
**11**	**No. 11**	**84.87 ± 0.79**	**9.98**
12	No. 12	-5.56 ± 2.87	-
13	No. 13	8.48 ± 1.75	-
14	No. 14	20.57 ± 3.55	-
15	No. 15	4.95 ± 0.85	-
**16**	**No. 16**	**75.32 ± 2.68**	**18.37**
17	No. 17	6.40 ± 1.20	-
18	No. 18	30.77 ± 3.63	-
**19**	**No. 19**	**78.76 ± 1.80**	**15.62**
20	No. 20	-3.73 ± 3.68	-
21	No. 22	-27.62 ± 3.70	-
22	No. 24	1.90 ± 2.69	-
23	No. 27	-37.34 ± 1.22	-
24	No. 28	-13.50 ± 2.59	-
25	No. 31	-7.60 ± 1.49	-
26	No. 32	-25.86 ± 0.30	-
27	No. 34	-33.32 ± 0.54	-
28	No. 35	-32.28 ± 2.53	-
29	No. 36	-42.65 ± 1.55	-
30	No. 39	-28.90 ± 0.91	-
31	No. 43	-21.35 ± 3.40	-
32	No. 44	-21.69 ± 1.47	-
33	No. 45	-51.68 ± 3.32	-

Results show in a standard form of mean ± SD (standard deviation) from three independent determinations (N=3).
Bold indicates an active extract showing inhibitory activity of more than 70%.

Table S1. Anti-mushroom tyrosinase Screening of sixty-two Thai rejuvenating remedies with 80% ethanolic extracts. (continue)

No.	Thai rejuvenating remedies (Sample code)	Tyrosinase inhibitory activity	
		Tyrosinase inhibition at 20 µg/ml (%)	IC_{50} values (µg/ml)
34	No. 46	-28.28 ± 2.28	-
35	No. 47	-50.28 ± 3.15	-
36	No. 48	-55.75 ± 0.28	-
37	No. 49	-17.33 ± 0.44	-
38	No. 50	-44.81 ± 3.19	-
39	No. 51	-29.49 ± 1.47	-
40	No. 52	-1.29 ± 2.57	-
41	No. 53	-7.14 ± 1.16	-
42	No. 54	-12.11 ± 2.16	-
43	No. 55	-8.23 ± 1.22	-
44	No. 57	-8.66 ± 0.61	-
45	No. 58	-13.86 ± 1.30	-
46	No. 59	-21.95 ± 1.10	-
47	No. 60	-44.47 ± 3.18	-
48	No. 61	0.49 ± 2.63	-
49	No. 62	-39.40 ± 0.16	-
50	No. 63	-30.49 ± 0.53	-
51	No. 64	-7.47 ± 2.53	-
52	No. 65	-3.04 ± 2.36	-
53	No. 66	-2.05 ± 2.91	-
54	No. 67	7.01 ± 1.87	-
55	No. 68	-14.20 ± 1.40	-
56	No. 70	-17.51 ± 3.64	-
57	No. 71	-5.71 ± 3.20	-
58	No. 72	-9.90 ± 0.63	-
59	No. 73	-11.19 ± 2.43	-
60	No. 74	41.19 ± 1.89	-
61	No. 75	-29.24 ± 2.94	-
62	No. 76	-14.36 ± 0.54	-
***Kojic acid*[P]**		***86.77 ± 2.97***	***3.08***
***Artocarpus lakoocha wood, water extract*[P]**		***95.12 ± 0.49***	***0.07***

Results show in a standard form of mean ± SD (standard deviation) from three independent determinations (N=3). Bold indicates an active extract showing inhibitory activity of more than 70%.

Table S2. Intercellular melanin content of B16F1 cells treated with α-MSH, positive controls (arbutin, kojic acid, and *A. lakoocha* wood, water extract), and test samples (crude, hexane, ethyl acetate, and ethanol extracts of the selected remedy)

	Melanin contents	SD	%Reduction
**Control**	34.8	1.91	-
**Arbutin**	18.67	0.50	46.35
**Kojic acid**	27.27	1.26	21.64
A. lakoocha* extract	30.29	0.68	12.96
**Crude extract**	25.85	0.94	25.72
**Hexane extract**	21.67	1.07	37.73
**Ethyl acetate extract**	18.49	1.02	46.87
**Ethanol extract**	33.26	1.10	4.43
**Water extract**	30.85	1.18	11.35
G. glabra* extract	28.57	1.12	17.90
**Glabridin***	9.56	0.66	72.53

* indicates samples tested in 5 μg/ml concentration

Table S3. Cell viability test of arbutin up to 200 μg/ml concentration.

Arbutin	5 μg/ml	10 μg/ml	50 μg/ml	100 μg/ml	150 μg/ml	200 μg/ml
**Average**	111.97	109.30	104.02	97.41	92.03	86.53
**SD**	4.55	14.08	5.16	1.44	4.32	8.79

Table S4. Anti-zebrafish larvae pigmentation of positive controls (1-phenyl-2-thiourea, PTU), Thai rejuvenating remedy No. 11 extracts (crude, hexane, ethyl acetate, ethanol, and water extracts), and active ingredients (*G. glabra* and glabridin) at 10 μg/ml concentration. 0.03% sea salt solution is used as a negative control.

	Area of black spot	SEM	% Inhibition
**Control**	22527	2104.2	
**PTU**	11852	608.15	47.39
**Crude extract**	26382	2195.6	-17.11
**Hexane extract**	24118	2201.2	-7.06
**Ethyl acetate extract**	23805	2563.8	-5.67
**Ethanol extract**	19171	2873.9	14.90
**Water extract**	21143	2061.3	6.14
***G. glabra* extract**	23945	2195.6	-6.29
**Glabridin***	30622	3655.9	-35.93

Table S5. Accession numbers of all tyrosinase amino acid sequences used in this study.

Name	Accession number
appleTYR	Q93XM8
riceTYR	A5X3J7
moldTYR	I7ZSX1
mrTYR	C7FF04
bovineTYR	Q8MIU0
bovineTYRP1	Q8WN57
bovineTYRP2	Q95119
hTYR_model	P14679
hTYRP1	P17643
hTYRP2	P40126
muTYR	P11344
muTYRP1	P07147
muTYRP2	P07147
goldenfishTYR	C6YBD2
salmonTYR	Q19VI0
zfTYR	F1QDZ4
zfTYRP1a	F1QEC9
zfTYRP1b	Q6DGE4

Table S6s. Chemical list in the PubChem database.

Name	CID number
Glabridin	124052
3′′,4′′-Dihydroglabridin	Self-made
Morusone	Self-made
Ethyl p-methoxycinnamate	5281783
3-Methyl-4-hydroxycinnamic acid	53404339
Caffeic acid	689043
